# The Reversal Intervention for Metabolic Syndrome (TRIMS) study: rationale, design, and baseline data

**DOI:** 10.1186/1745-6215-12-107

**Published:** 2011-05-04

**Authors:** Alison J Dunkley, Melanie J Davies, Margaret A Stone, Nicholas A Taub, Jacqui Troughton, Thomas Yates, Kamlesh Khunti

**Affiliations:** 1 Department of Health Sciences, University of Leicester. Leicester, UK; 2 Department of Cardiovascular Sciences, University of Leicester, Leicester, UK; 3 University Hospitals of Leicester NHS Trust, Leicester, UK

## Abstract

**Background:**

Recent attention has focused on strategies to combat the forecast epidemic of type-2 diabetes (T2DM) and its major vascular sequelae. Metabolic syndrome (MetS) comprises a constellation of factors that increase the risk of cardiovascular disease (CVD) and T2DM. Our study aims to develop a structured self-management education programme for people with MetS, which includes management of cardiovascular and diabetes risk factors, and to determine its impact. This paper describes the rationale and design of the TRIMS study, including intervention development, and presents baseline data.

**Methods:**

Subjects recruited from a mixed-ethnic population with MetS were randomised to intervention or control arms. The intervention arm received structured group education based on robust psychological theories and current evidence. The control group received routine care. Follow-up data will be collected at 6 and 12 months. The primary outcome measure will be reversal of metabolic syndrome in the intervention group subjects compared to controls at 12 months follow-up.

**Results:**

82 participants (44% male, 22% South Asian) were recruited between November 2009 and July 2010. Baseline characteristics were similar for both the intervention (n = 42) and control groups (n = 40). Median age was 63 years (IQR 57 - 67), mean waist size 106 cm (SD ± 11), and prescribing of statins and anti-hypertensives was 51% in each case.

**Conclusion:**

Results will provide information on changes in diabetes and CVD risk factors and help to inform primary prevention strategies in people with MetS from varied ethnic backgrounds who are at high risk of developing T2DM and CVD. Information gathered in relation to the programme's acceptability and effectiveness in a multi-ethnic population would ensure that our results are widely applicable.

**Trial registration:**

The study is registered at ClinicalTrials.gov, study identifier: NCT01043770.

## Background

The predicted global increase in type-2 diabetes (T2DM) and cardiovascular disease (CVD), and associated morbidity and mortality, are a growing public health burden [1,2]. This is largely due to rising levels of obesity, and sedentary lifestyles. Additionally, the disease burden in certain ethnic groups such as South Asians (SAs) is significantly higher than for White Europeans (WEs) [3,4]. Strategies to reduce the development of CVD and T2DM in high risk individuals are a priority.

People who are centrally obese often have a clustering of additional cardiovascular and diabetes risk factors such as elevated blood pressure, dyslipidaemia and impaired glucose metabolism, which have been linked to insulin resistance and collectively termed metabolic syndrome (MetS) [5,6]. Prevalence estimates for MetS vary widely between populations [7]. It has previously been suggested that between 13 - 30% of people in developing countries have MetS [8] and around 35% in high-income nations such as the US [9]. Additionally, the prevalence of MetS increases with age, is higher in socio-economically disadvantaged groups [10,11], and shows ethnic differences [12]. UK data suggest that approximately 30 - 34% of adults aged 40 to 75 years have MetS [13], and this number is likely to rise as lifestyles continue to become increasingly inactive.

Lack of a consensus definition and concerns about whether the risk conveyed by the syndrome as a whole exceeds the total risk associated with individual components, have led to debate about the prognostic significance of MetS [14]. Its usefulness in clinical practice compared to existing tools such as the Framingham CVD risk score [15,16], has also been questioned. However, MetS is linked to an increased risk of developing both CVD and T2DM. Evidence indicates that people with MetS are approximately twice as likely to have CVD (incident disease or event) [17], and are between 3.5 to 5 times as likely to develop T2DM [18]. Furthermore, MetS is of great importance to public health as it precedes T2DM and CVD by several years. People with MetS could, therefore, be an important group to target for primary prevention of T2DM and CVD [14,19]. Additionally, MetS could be a useful concept for healthcare professionals and patients to focus on when addressing the health risks associated with abdominal obesity [20]. It is therefore essential to develop a pragmatic early intervention that can be easily implemented to a large number of people in primary care.

A recent meta-analysis suggests that intensive lifestyle programmes targeted at people with pre-diabetes, who are at high risk of T2DM, are effective in reducing the incidence of diabetes by more than 50% [21]. However, targeting dysglycaemia in isolation may not be the best approach if the ultimate aim is to reduce the rate of cardiovascular complications. Evidence from clinical trials is limited regarding the effectiveness of strategies aimed at primary prevention of T2DM and/or CVD in people with MetS. Research is therefore needed into the efficacy of lifestyle interventions for primary prevention of T2DM and CVD that can be applicable to multi-ethnic populations with MetS.

The aim of The Reversal Intervention for Metabolic Syndrome (TRIMS) study is to investigate the hypothesis that delivery of a group self-management education programme designed to encourage lifestyle changes in individuals identified with MetS would be a feasible, acceptable, and effective strategy for primary prevention of CVD and T2DM. Specific objectives of the study are to: a) develop an evidence based education programme to improve cardiovascular risk and dysglycaemia in people with MetS in primary care; b) determine the impact of attending an education programme on features of MetS and quality of life after 12 months of follow-up; and c) assess acceptability, uptake, and feasibility of implementing a group self-management education programme in an ethnically diverse population of individuals with MetS.

Development, evaluation and implementation of the TRIMS intervention is guided by the most recent Medical Research Council (MRC) Framework for developing and evaluating complex interventions [22]. This article outlines the development, piloting and initial exploratory evaluation of the study intervention. Additionally, the paper describes the design of the main TRIMS study, including the methods used for delivering the trial intervention and for carrying out a definitive evaluation. Baseline data are also reported.

## Methods

### Study design

The TRIMS study was designed as a single-centre, 2 arm, parallel, 12 month randomised controlled trial (RCT) that compared the effectiveness of structured group lifestyle education (intervention) with usual care (control). Prior to commencing the main trial, an in-depth development and feasibility phase was carried out to ensure that the design of the intervention was appropriate for people with MetS.

#### 1. Development and feasibility phase

##### Curriculum development

We adapted an existing group lifestyle education programme (Let's Prevent) which was originally developed for people with pre-diabetes [23]. The style, content and process of this programme draw on a range of concepts from health psychology and education [24-26] (see Table 1) and its philosophy is centred on patient empowerment. The key nutritional and physical activity messages are based on evidence from previous diabetes prevention trials [27-29]. Permission to adapt the pre-diabetes programme was granted by the DESMOND collaborative [30]. Using this curriculum as a framework, the TRIMS curriculum was revised to make it more suitable for people with MetS, who may or may not be dysglycaemic, and include additional emphasis on management of cardiovascular risk.

**Table 1:** Main theories underpinning the structured group education self-management approach

Theory	Key elements
**Common sense model**	People tend to conceptualise a health threat/problem according to 5 domains:
	Identity; Cause; Timeline; Consequences; Control/cure
	Important to elicit these beliefs as thought to influence coping and self-care behaviour
	Influenced by social and cultural factors
	Health information needs to be aimed at targeting all 5 domains. If not:
	Individual is likely to acquire the missing information from another source
	Risk of forming spurious health beliefs
	Could negatively impact subsequent coping behaviour

**Dual process theory**	Systematic processing of information is encouraged
	Individual's are encouraged to take an active role in their learning and work things out and ask questions
	The educator does not lecture or dictate but uses open questions to elicit information
	Active learning requires recipient to make more effort. However:
	Results in individual's making a stronger link between theoretical concepts and their personal situation
	Attitude change generally lasts longer when produced by systematic processing

**Social cognitive (learning) theory**	Behavioural change is influenced by an individual's:
	Sense of control or perceived self-efficacy
	Expectancies about outcomes of personal actions
	Social modelling of knowledge and competencies
	People learn from interaction with others.
	Helps a person to realise what they already know
	Cultivates new competencies
	Instils behavioural outcome expectations
	The educator supports individuals to put the elements in place and move forward

References: Common sense model [26]; Dual process theory [25]; Social cognitive (learning) theory [24]

A multi-faceted approach was adopted to inform the detailed development of the TRIMS education programme. Firstly, existing evidence regarding the effectiveness of lifestyle interventions for reversing metabolic syndrome was collated by conducting a systematic review and meta-analysis [31]. Secondly, currently published guidelines and recommendations were reviewed, some specifically for MetS and others providing more general guidance related to management of CVD risk, and diet and nutrition [5,6,32-39]. A combined approach was used to ensure that the lifestyle behaviour modifications recommended by the TRIMS programme were evidence based and also in line with current UK practice guidance. The evidence to support specific lifestyle elements of the programme is outlined in Table 2.

**Table 2:** Key lifestyle elements included in the TRIMS education programme

Modifiable lifestyle factors	Key lifestyle elements	Source of evidence		
		
		MetS systematic review	MetS specific guidelines & recommendations	Other guidelines & recommendations
PHYSICAL ACTIVITY	Increase physical activity	✓	✓	✓

WEIGHT/WAIST SIZE	Sustained weight loss	✓	✓	✓
	Moderate calorie restriction	✓	✓	✓

DIETARY FACTORS	Dietary consumption:			
				
Fibre	Increase wholegrains (reduce refined carbohydrates)	✓	✓	✓
	Increase legumes	✓	✓	✓
	Increase fruit and vegetables	✓	✓	✓
				
Fats	Moderate reduction in total fat	✓	✓	✓
	Reduce saturated fat	✓	✓	✓
	Increase mono-unsaturated fat	✓	✓	✓
				
Omega-3	Increase fish/oily fish	✓	✓	✓
				
Salt	Reduce salt	✓	✓	✓
				
Alcohol	Alcohol in moderation	✓	✓	✓
				

SMOKING	Cessation of smoking	✓	✓	✓

References: MetS systematic review [31]; MetS specific guidelines & recommendations [5,6,37,38]; Other guidelines & recommendations [32-36].

Subsequently, an initial curriculum was developed by a multi-disciplinary team. Additional resources were also developed including a handbook for patients to reinforce what they had learnt on the course and use as a resource in the future. The main aims of the TRIMS programme were: to increase knowledge and understanding of MetS, including raising awareness of possible future health risk and of potential prevention strategies; to enhance self-efficacy and self-regulatory skills in order to promote healthy lifestyle behaviours. The key behavioural goals the programme aimed to promote included: 1) increasing physical activity, ≥ 45 minutes of moderate intensity activity (or an extra 4,500 steps) per day; 2) losing weight (reducing waist size), a reduction of between 5 - 10% of initial body weight through increasing physical activity and/or reducing calorie intake; 3) increasing dietary fibre consumption, particularly wholegrains, legumes, fruits and vegetables; 4) reducing consumption of saturated fats; 5) reducing salt intake; 6) increasing intake of omega-3 fatty acids; and 7) smoking cessation. However, despite suggested targets, the emphasis of the programme was on enabling participants to set their own realistic personalised goals for behaviour change.

##### Piloting

Permission to pilot the education sessions within primary care was obtained from Leicester City Primary Care Trust. One large general practice in the city was subsequently approached and agreed for their patients to be involved. From this practice a sample of patients who were on the hypertension register and also met the criteria for MetS were sent a letter and information sheet by their general practitioner (GP), identifying them as having MetS and inviting them to attend the education sessions. This was followed-up by a telephone call from their GP approximately one week later.

The structured group education programme was delivered as two 3-hour, afternoon sessions held 2 weeks apart, at the health centre where the GP practice was based. Two experienced health educators, a nurse and a dietician, led the sessions. The sessions were designed to encourage participation and included games/activities. Participants were also supported to identify personal risk factors that they wished to change and to formulate a self-management plan. As part of the education sessions the participants were provided with a pedometer and activity logbook to use as motivational tools, and a handbook to use during sessions and then take away as a resource in the future.

A range of methods was used to evaluate the education sessions and collect feedback. These included observations recorded during the sessions by an experienced researcher, reflections from the two educators leading the programme, and semi-structured interviews conducted by telephone with volunteers who had attended the education sessions. Qualitative data from these sources were collated using Framework Charting [40].

Observations and feedback indicated that, at the start of the education programme, people found it difficult to comprehend MetS as it was an unfamiliar concept. However, with repeated explanations and reinforcement throughout the programme, the sessions helped individuals to understand the syndrome, including the role of abdominal obesity (waist size) and possible future health risks. This was something people said that they valued and it enabled them to focus on their own personal risk. Key messages that people felt they had taken away from the sessions included: making healthier food choices, reading food labels and using a pedometer and log-book to help increase activity levels. For some parts of the curriculum, some individuals expressed a preference for a more direct approach, with the educator talking more and less group discussion, specifically for topics with which people were less familiar. Overall, learning as part of a group was favoured by individuals as they felt they benefitted from the questions that other people asked and the sharing of experiences.

##### Modifications to the intervention as a result of piloting

Findings from the pilot of the intervention were used to help refine the curriculum, resources and style of delivery of the programme. The content and structure of the final TRIMS education programme used for the main trial is outlined in Table 3. Revisions made after piloting included changes to the introduction section at the start of the education programme, to ensure that educators made participants aware of the non-didactic approach that would be used. This included emphasising the benefits of the style of delivery to be used, including group participation and facilitation of learning through the use of open questions, games and activities. This reinforced explanations given in a pre-course booklet that introduced people to what they could expect when they attended the group sessions. Another revision to the programme involved simplifying the way in which MetS was explained. The part of the curriculum related to understanding MetS, particularly the sections focusing on how the body uses and stores energy from food (fats and carbohydrates) and the role of insulin resistance, was amended. Additional prompts were also added, to help educators to link explanations about MetS to any prior perceptions and beliefs that participants may have shared as part of the patient story section. The accompanying resources were also modified. Additionally, in response to feedback, a food diary was added to the participant handbook so that people could optionally record their daily food intake. This complemented the physical activity logbook already provided.

**Table 3:** Outline plan of the TRIMS education programme

PART 1 - First week	Overview of the main aims and activities	Theory
**A: Introduction and Housekeeping **(5 minutes)	To inform participants of the aims of the course, main topics to be covered, and the style of delivery	

**B: The Patient Story **(20 minutes) 1) Names 2) How did you find out you had metabolic syndrome 3) What do you think it is? Causes? 4) What will it mean for my health? Treatments? 5) Have you a question?	To elicit an individual's experiences, perceptions and health beliefs Participants are encouraged to share their experiences and beliefs with the rest of the group, and identify any questions they have	Common sense model

**C: Metabolic Syndrome and Insulin Resistance** 1) Understanding metabolic syndrome (55 minutes) • Energy from food - food groups • Healthy metabolism - energy used/stored • Abdominal obesity and insulin resistance • Cholesterol • Blood pressure 2) How does metabolic syndrome affect me? (40 minutes) • Understanding your personal results • Causes • Reversing metabolic syndrome and reducing the risk of T2DM and CVD	To help participants understand what metabolic syndrome is, possible causes, what it means to their health, and possible ways to reduce their future health risk Participants are helped to work through what is happening in the body with metabolic syndrome, complete their own personal health profile, and consider how they were identified as having metabolic syndrome	Common sense model Dual process theory

**D: Physical Activity **(40 minutes) 1) Benefits 2) Recommendations 3) Measuring activity 4) Barriers and facilitators	To facilitate exploration of the recommendations and benefits of physical activity, and possible barriers Participants are: Encouraged to consider ways to increase their activity levels (including their own personal activity targets)and how this could reduce future health risk Shown and discuss how they can use a pedometer and logbook as a motivational tool and encouraged to go away and use them before their next session	Common sense model Dual process theory Social cognitive (learning) theory

**E: How Am I Doing**	Participants are encouraged to reflect on the main messages so far and start to think about possible lifestyle changes	Social cognitive (learning) theory

**PART 2- Second week**		

**F: Reflections **(5 minutes)	Participants are encouraged to reflect on issues that have come up and share these with the group	Social cognitive (learning) theory

• **G: Weight management and Food Choices (1) **(35 minutes) 1) Factors influencing food choices 2) Monitoring weight/shape 3) Energy balance 4) Losing weight/reducing waist size 5) Food messages • Fat, alcohol, fruit and vegetables	To help participants explore factors involved in weight management, and consider food choices. Participants are: Encouraged to consider practical ways to lose weight/reduce waist size Helped to work through what factors can cause changes in weight and which foods are higher sources of calories Shown and discuss how they could use a food diary to record what they eat and drink and identify possible changes they could make.	Common sense model Dual process theory Social cognitive (learning) theory

**H: Food Choices (2) **(75 minutes) 1) Types of fats 2) Omega-3 3) Fibre 4) Salt 5) Making healthier food choices	To facilitate exploration of the recommendations and benefits of making healthier food choices, and how these relate to metabolic syndrome and individual risk factors Participants are encouraged to consider ways to make healthier food choices (including reading food labels) and how this could reduce future health risk	Common sense model Dual process theory Social cognitive (learning) theory

**I: Metabolic Syndrome Self-Management Plan **(40 minutes) 1) Additional risk factors - smoking, depression 2) Behaviour change 3) Identifying personal risk factors & completing an action plan	To help participants to identify a behavioural goal they can aim for to improve their risk profile/reverse metabolic syndrome, and make a realistic plan of action for this behaviour change Participants are helped to: Identify things they want to change based on their personal health profile, Explore possible options utilising information from previous sessions Identify personal barriers Develop their own personal action plan	Social cognitive (learning) theory

**J: Questions and Future Care **(10 minutes)	To ensure that all questions previously raised by participants have been answered fully, and that they know how to access ongoing care and support	Common sense model

Throughout the sessions a combination of open questions, analogies, visual aids, games, activities and a personal handbook are used by the educators to assist participants learning. Each week included a 15 minute break.

References: Common sense model [26]; Dual process theory [25]; Social cognitive (learning) theory [24]

#### 2. The TRIMS randomised controlled trial

##### Participant recruitment

Local ethics and research governance approvals were obtained prior to conducting the main TRIMS RCT. General practices (in Leicestershire, UK) who were already taking part in local population based diabetes screening studies [41-43] were approached to participate. Potential participants aged 40 - 74 years, from volunteer practices, were then recruited by postal invitation using two different recruitment strategies. Firstly, eligible people identified as having MetS (International Diabetes Federation (IDF) definition [6]) according to their previous screening results, were sent a postal invitation via their GP. Secondly, people who had consented to be approached with details of other research studies, when they participated in a screening study, and who met the inclusion criteria were sent a letter of invitation by the principal investigator of the screening study. Exclusion criteria included previous history of T2DM or CVD; pregnancy and/or breast feeding; life-limiting terminal illness; lack of capacity to give informed consent; being housebound or residing in a nursing/care home; and inability to understand, speak and read English.

Respondents were asked to attend for an initial appointment having fasted overnight for at least 8 hours. Written informed consent was obtained from volunteers by a research nurse prior to carrying out any tests or measurements. Participants underwent a 75 g oral glucose tolerance test (OGTT) [44], and had additional demographic and bio-medical data collected in a standardised way, according to the schedule in Table 4. Data collected included measurements to confirm eligibility and MetS status; however, if relevant blood tests for glucose and lipids had been conducted for screening within the last 3 months these were not repeated and the initial screening values were used as the baseline values.

**Table 4:** Data collection schedule and outcome measures

Collected by research nurse	Baseline	6 months	12 months
Bloods & biomedical data			
FPG	✓	✓	✓
2 hour glucose	✓	✓	✓
HbA1c	✓	✓	✓
Total cholesterol	✓	✓	✓
HDL	✓	✓	✓
Triglycerides	✓	✓	✓
Albumin:creatinine ratio (urine)	✓	✓	✓
Blood pressure	✓	✓	✓
Waist circumference	✓	✓	✓
Hip circumference	✓	✓	✓
Height	✓	✗	✗
Weight	✓	✓	✓
Medical history	✓	✓	✓
Current medication	✓	✓	✓
Smoking status	✓	✓	✓
Biomarkers			
Insulin, Hs-CRP, Adiponectin	✓	✗	✓
Demographic details			
Age	✓	✗	✗
Sex	✓	✗	✗
Ethnicity *(adapted from classification used for 2001 UK census)*	✓	✗	✗
Current employment status *(working, retired, unemployed, long term sick/disabled, never worked, other)*	✓	✗	✗
Education *(age finished full time education, & highest level of qualification held)*	✓	✗	✗
Socio-economic classification *(NS-SEC-5-Class) *[57]	✓	✗	✗
Deprivation score *(IMD score, 2007)*[58]	✓	✗	✗

**Self-reported data**			

Questionnaires			
Physical activity *IPAQ (short form) *[47]	✓	✓	✓
Anxiety & depression *HADS *[48]	✓	✓	✓
Quality of life *EQ-5D *[49]	✓	✓	✓
Dietary habits *DINE *[50]	✓	✓	✓
General self-efficacy *GSE*[51]	✓	✓	✓

**Other**			

Ambulatory activity	✓	✓	✓

Abbreviations: **FPG **(fasting plasma glucose); **HbA1c **(glycated haemaglobin); **HDL **(high density lipoprotein cholesterol); **NS-SEC-5-Class **(National Statistics Socio-economic Classification 5-Class version); **IPAQ **(International Physical Activity Questionnaire); **HADS **(Hospital Anxiety & Depression Score); **EQ-5D **(EuroQol EQ-5D questionnaire); **DINE **(Dietary Instrument for Nutrition Education); **GSE **(General Self Efficacy); **IMD **(Indices of Multiple Deprivation)

##### Randomisation

Following their baseline appointment, eligible volunteers were randomised to either the study intervention arm (routine care plus TRIMS group education programme) or control arm (routine care) using computer-generated block randomisation. The researcher who held the randomisation sequence had no involvement with the recruitment of participants or baseline data collection. Blinding of participants was not possible due to the nature of the study. Participants were informed of their group allocation by a letter sent in the post and volunteers GPs were also provided with this information. People in the intervention group were informed that they would be given their bio-medical results as part of the TRIMS education and the control group were asked to contact their GP if they wanted any information about their results.

###### Delivery of the TRIMS intervention

Intervention group participants were contacted by telephone within 1 - 2 weeks of their baseline assessment and invited to attend the TRIMS education programme within the following 2 months. The education sessions were held Monday to Saturday, at various local community venues and consisted of 6 hours of contact time spread over two 3 hour sessions, held approximately 2 weeks apart. Participants were also invited to bring a friend or relative for both social and practical support. Two trained educators (AJD plus another nurse or a dietician) facilitated the groups. The approximate proportions of the curriculum devoted to specific topics are outlined in Table 3. In addition to attending the initial group education, at 6 months people will be given the option of receiving additional support from an educator (AJD), via the telephone, to answer any queries and concerns and to help participants update their self-management education resources.

###### Primary and secondary outcomes

The primary outcome is reversal of MetS according to the IDF criteria in the intervention group compared to the control group, after 12 months of follow-up. Taking into account the ethnic diversity within our target population, the IDF criteria were chosen in preference to the National Cholesterol Education Panel (NCEP) definition due to the provision of ethnic specific cut points for waist circumference and central obesity (Table 5). Secondary outcomes compared at baseline versus 12 months, and for the intervention group versus the control group, see Table 4, include changes in: i) the prevalence of MetS according to NCEP criteria; ii) individual components of the MetS (fasting plasma glucose, triglycerides, high density lipoprotein cholesterol (HDL), blood pressure, waist circumference), and 2 hour glucose.

**Table 5:** The International Diabetes Federation (IDF) and updated National Cholesterol Education Panel (NCEP) definitions of metabolic syndrome.

**NCEP (2005)**[37]	**IDF (2005)**[6] ESSENTIAL REQUIREMENT **Central obesity** **Waist circumference** >94 cm^† ^(males) >80 cm^† ^(females)
*ANY 3 out of the following 5* ↓	***+ ****ANY 2 out of the following 4* ↓
**1) Raised fasting plasma glucose:** ≥ 5.6 mmol/l*	**1) Raised fasting plasma glucose:** ≥ 5.6 mmol/l
2) Raised triglycerides:** ≥ 1.7 mmol/l and/or 3) Low HDL cholesterol:** < 1.03 (males) < 1.29 (females)	**2) Raised triglycerides:** ≥ 1.7 mmol/l *(or specific treatment for)* and/or **3) Reduced HDL cholesterol** < 1.03 (males) < 1.29 (females), *(or specific treatment for)*
4) Hypertension:** Blood pressure ≥ 130/85 mmHg	**4) Raised blood pressure:** Blood pressure ≥ 130/85 mmHg (or treatment for previously diagnosed hypertension)
**5) Central obesity:** Waist circumference > 102 cm (males) > 88 cm (females)	
*Updated from the original NCEP 2001 definition *[5]: *previous fasting plasma glucose value (≥6.1 mmol/l) was updated to include a lower value; ** was updated to include drug treatment .	*^†^Waist circumference ethnic-specific for Europid men & women; Waist circumference for South Asians (Chinese, Malay, & Asian Indian): ≥90 cm (males), & ≥80 cm (females)*.

###### Data collection and assessment of outcomes

Routine laboratory methods were used for all biochemical measurements. Serum total cholesterol, HDL cholesterol, and triglycerides; plasma fasting and 2-hour glucose; and urine albumin and creatinine, were measured using a Siemens Adiva 2400 analyser (Siemens Healthcare Diagnostics, Camberley, UK). Glycated haemoglobin (HbA1c) was measured using a Tosoh G7 analyser (Tosoh Bioscience Ltd, Redditch, UK). Low density lipoprotein cholesterol (LDL) was estimated using the Friedewald equation[45]. If participants gave consent, additional blood was taken at baseline (and 12 months) for measurement of bio-markers that are linked to MetS (high-sensitivity- C-reactive protein (hs-CRP), adiponectin and insulin)[46]. After processing, the serum for bio-markers was stored in aliquots at -80°c and these samples will be analysed as a single batch at the end of the study.

Resting blood pressure was measured using an Omron automatic blood pressure monitor, (Omron Healthcare UK Ltd) and a mean value was calculated from the last 2 measurements in a series of three. Waist circumference was measured midway between the costal margin and the iliac-crest, in the mid-axillary line, over minimal clothing and at the end of expiration, and was recorded to the nearest mm. Hip circumference was measured at the widest point over the buttocks and to the nearest mm. Weight in light clothing and no shoes was recorded to the nearest 0.1 kg using a digital scale, and height to the nearest cm using a stadiometer and with head placed in the Frankfurt plane. Additional data were collected on physical activity [47], anxiety and depression [48], quality of life [49], dietary habits [50] and self-efficacy [51] using validated questionnaires that were self-completed by participants, as outlined in Table 4. Ambulatory activity was measured using a CW-700 Yamax Digi-Walker electronic pedometer with a 7-day memory (Yamax Corporation, Tokyo, Japan) and an average step count per day was calculated from measurements from at least 3 days.

#### Follow-up data collection

The control and intervention groups will be recalled at 6 months and 12 months for repeat measurements (Table 4). All persons involved in the collection of follow-up data will be independent and blinded to study group allocation.

##### Feasibility and acceptability

Acceptability of the TRIMS education programme will be measured by obtaining qualitative feedback. Possible topics to be explored include ease of understanding; views about the content of the programme and style of delivery; and usefulness and relevance (including cultural relevance). An independent researcher will conduct semi-structured interviews via the telephone. Information about the interviews and a reply slip will be given in person or posted to participants who attend the education sessions. Purposive sampling will be used to select a demographically varied sample of subjects to be interviewed from those who volunteer. Written consent will be obtained in advance by post and confirmed verbally at the time of the interview. Feasibility will be assessed through identification and consideration of problems encountered during implementation of the intervention and uptake will be measured by comparing the number of responses to the number of invitations.

##### Sample size

The TRIMS study is powered to detect a between group difference of 30% in the proportion of people with prevalent MetS at 12 months (prevalence of MetS reduced to 60% in the intervention group and 90% in controls, alpha = 0.05, power = 0.80) and allowing 20% for loss to follow up, 80 participants were required in total, 40 people in each of the control and intervention arms. The power calculation was based on the results of the Oslo Diet and Exercise Study (ODES)[52] which achieved a difference of 55% between the diet and exercise group and the control group. With our less intensive group lifestyle programme a more modest difference of 30% was assumed.

##### Data analysis

Continuous outcome variables were tested for normality and independent-sample t-tests or Mann Whitney-U tests were used to compare between group differences at baseline; chi-square tests were used to compare categorical variables. Baseline data entry was conducted blind to group assignment, and steps were taken to ensure that data analysis was blinded as far as possible. Analysis of follow-up data will be conducted on an intention to treat basis. The study groups will be compared with respect to proportions of subjects with MetS at 12 months using the chi-squared test, followed by logistic regression modelling to adjust for any substantial chance imbalance between the groups, including adjustments for gender and ethnicity. Further study outcomes will be compared using similar methods and linear modelling, as appropriate and 95% confidence intervals will be calculated for treatment effects, corresponding to the statistical testing. Significance will be assessed at the 5% level. PASW Statistics version 18.0 (SPSS Inc.) will be used to conduct all statistical analyses. Qualitative feedback collected in order to gauge the acceptability of the TRIMS programme will be analysed using a thematic approach including the use of charting [40] to collate the data.

## Results

**Figure 1:**
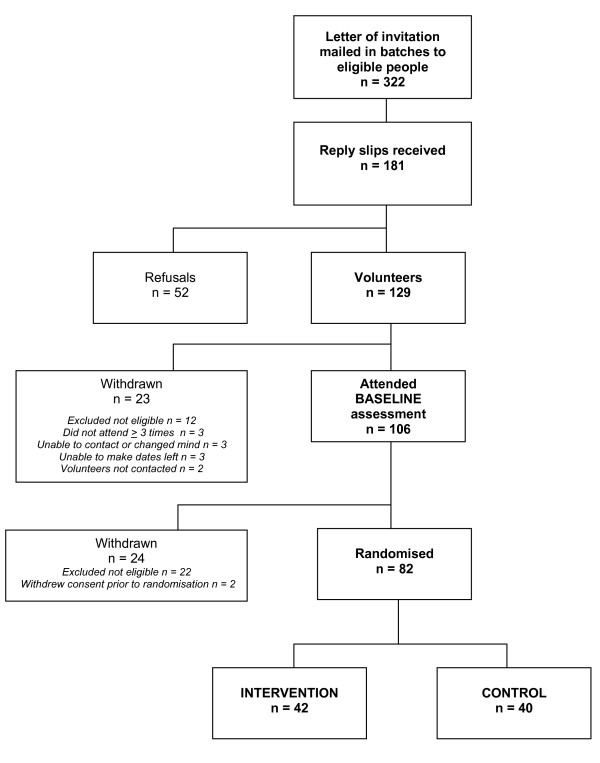
**Flow chart of recruitment and randomisation to TRIMS Study**.

Participants were recruited to the main trial between November 2009 and July 2010 (Figure 1). In total, 322 potentially eligible people were invited to participate from 8 different general practices. Of those people who were invited, 40% (n = 129) volunteered to participate, 16% (n = 52) refused, and 44% (n = 141) did not reply. Reasons given by those who declined to participate (n = 52) included lack of time due to work or other commitments (23%), no perceived need for additional advice or health checks (29%), and other health problems (5%). Overall, 82 people were enrolled onto the study, 42 to the intervention group and 40 to the control.

Table 6 shows the key characteristics for the overall study population and by treatment group. The randomisation procedure led to balanced samples in the intervention and control groups, with no statistically significant differences in the demographic or clinical characteristics between the study arms. Of the 82 participants, 36 (44%) were male, 18 (22%) were of SA ethnicity, median age was 63 years (IQR 57 - 67), mean waist size 106 cm (SD ±11), BMI 30 kg/m^2 ^(IQR 28 - 33), 10 (12%) were current smokers, and 42 (51%) of participants were prescribed a statin and 42 (51%) an anti-hypertensive.

**Table 6:** Key baseline characteristics of participants for all individuals and by study group

Characteristics	n	All (n = 82)	n	Intervention (n = 42)	n	Control (n = 40)	p value (I vs. C)
Demographic characteristics							
**Age (yrs)**	*82*	63 [57 - 67]	*42*	65 [55 - 68]	*40*	62 [58 - 67]	p = 0.831
**Sex: ***Male*	*82*	36 (43.9)	*42*	21 (50.0)	*40*	15 (37.5)	p = 0.359
**Ethnicity:**	*82*		*42*		*40*		p = 0.268
*South Asian*		18 (22.0)		11 (26.2)		7 (17.5)	
*White*		62 (75.6)		31 (73.8)		31 (77.5)	
*Other*		2 (2.4)		0 (0.0)		2 (5.0)	
**IMD score (2007)**	*82*	19.50 [14.04 - 33.74]	*42*	21.49 [14.04 - 21.49]	*40*	18.59 [13.55 - 32.96]	p = 0.344
**NS-SEC-5 class:**	*82*		*42*		*40*		p = 0.708
*1*		17 (20.7)		9 (21.4)		8 (20.0)	
*2*		19 (23.2)		7 (16.7)		12 (30.0)	
*3*		10 (12.2)		6 (14.3)		4 (10.0)	
*4*		15 (18.3)		8 (19.0)		7 (17.5)	
*5*		21 (25.6)		12 (28.6)		9 (22.5)	

Measures related to MetS							
**Waist circumference(cm)**	*82*	105.5 ± 10.8	*42*	103.9 ± 10.2	*40*	107.2 ± 11.2	p = 0.161
**Systolic BP (mmHg)**	*82*	132.4 ± 15.4	*42*	134.9 ± 13.2	*40*	129.8 ± 17.3	p = 0.139
**Diastolic BP (mmHg)**	*82*	85.9 ± 9.5	*42*	85.6 ± 9.7	*40*	86.1 ± 9.5	p = 0.803
**HDL cholesterol (mmol/l)**	*82*	1.20 [1.00 - 1.50]	*42*	1.20 [1.00 - 1.53]	*40*	1.20 [1.03 - 1.48]	p = 0.367
**Triglycerides(mmol/l)**	*82*	1.60 [1.20 - 2.00]	*42*	1.45 [0.98 - 1.93]	*40*	1.60 [1.40 - 2.08]	p = 0.112
**FPG (mmol/l)**	*82*	5.21 ± 0.56	*42*	5.23 ± 0.44	*40*	5.19 ± 0.66	p = 0.714
**Proportion meeting IDF criteria for:**							
**BP**	*82*	73 (89.0)	*42*	37 (88.1)	*40*	36 (90.0)	p = 1.000
**FPG**	*82*	22 (26.8)	*42*	12 (28.6)	*40*	10 (25.0)	p = 0.908
**HDL**	*82*	72 (87.8)	*42*	35 (83.3)	*40*	37 (92.5)	p = 0.313
**Triglycerides**	*82*	67 (81.7)	*42*	35 (83.3)	*40*	32 (80.0)	p = 0.917
**Total n° of IDF criteria met:**	*82*		*42*		*40*		p = 0.924
*3*		25 (30.5)		13 (31.0)		12 (30.0)	
*4*		44 (53.7)		23 (54.8)		21 (52.5)	
*5*		13 (15.9)		6 (14.3)		7 (17.5)	

Other bio-medical measures							
**BMI (kg/m^2^)**	*82*	30.2 [28.1 - 33.1]	*42*	29.3 [27.8 - 32.2]	*40*	30.9 [28.8 - 34.1]	p = 0.140
**Total cholesterol (mmol/l)**	*82*	4.99 ± 0.86	*42*	4.82 ± 0.77	*40*	5.17 ± 0.93	p = 0.067
**LDL cholesterol (mmol/l)**	*80*	2.92 ± 0.74	*42*	2.80 ± 0.68	*38*	3.05 ± 0.80	p = 0.126
**HbA1c (mmol/l)**	*80*	42.0 [40.0 - 43.0]	*40*	41.0 [39.0 - 42.8]	*40*	42.5 [40.0 - 44.0]	p = 0.179
**2 hour glucose (mmol/l)**	*81*	5.80 ± 1.65	*41*	5.65 ± 1.43	*40*	5.95 ± 1.85	p = 0.419

Lifestyle & well being							
**Current smoker: ***yes*	*82*	10 (12.2)	*42*	4 (9.5)	*40*	6 (15.0)	p = 0.514
**Fruit & vegetables (portions/day)**	*82*	3.0 [2.0 - 4.0]	*42*	3.0 [2.0 - 4.0]	*40*	3.0 [2.0 - 5.0]	p = 0.429
**Pedometer counts (av steps/day)**	*72*	5762 [3365 - 8592]	*39*	6829 [3224 - 8596]	*33*	4774 [3522 - 8653]	p = 0.705
**EQ-5D score**	*82*	0.80 [0.72 - 1.00]	*42*	0.80 [0.73 - 1.00]	*40*	0.76 [0.69 - 1.00]	p = 0.293
**EQ-5D VAS**	*81*	80.0 [70.0 - 90.0]	*41*	80.0 [70.0 - 90.0]	*40*	80.0 [ 61.3 - 90.8]	p = 0.420

Parametric continuous data are expressed as mean ± S.D; nonparametric continuous data as median [IQR]; categorical data as number (%).

Other ethnicity category: 1 Caribbean Indian, 1 mixed Caribbean/White British. NS-SEC-5 class: 1 (Managerial & professional occupations); 2 (Intermediate occupations); 3 (Small employers & own account workers); 4 (Lower supervisory & technical occupations); 5 (Semi-routine & routine occupations).

IMD score: greater scores indicate a higher level of deprivation.

Abbreviations: **IMD **(Index of Multiple Deprivation); **NS-SEC **(National Statistics Socio-economic Classification); **BP **(blood pressure); **HDL **(high density lipoprotein cholesterol); **FPG **(fasting plasma glucose); **IDF **(International Diabetes Federation); **BMI **(body mass index); **LDL **(low density lipoprotein **); HbA1c **(glycated haemoglobin); **EQ-5D **(EuroQol EQ-5D); VAS (visual analogue scale).

According to pedometer measurements, the median number of steps/day for participants was 5762 (IQR 3365 - 8592). Self-reported time spent sitting was 300 mins/day (IQR 180 - 360). Dietary data indicated that 46% (n = 34) of people were classified as having a low fibre intake and 15% (n = 11) as having a high fat intake. The median number of portions of fruit, salad and vegetables consumed per day was 3 (IQR 2 - 4).

All participants had MetS according to the IDF definition: 31% (n = 25) met 3 criteria, 54% (n = 44) met 4 criteria, and 16% (n = 13) met 5 criteria. The MetS values for the individual criteria for BP, HDL and triglycerides were met by 80 - 90% of people. However, only 22 participants (27%), 12 (29%) intervention and 10 (25%) control, met the criterion for raised fasting plasma glucose. Overall, the prevalence of MetS according to the updated NCEP criteria[37] was 94% (n = 77), 93% (n = 39) intervention group and 95% (n = 38) control (p = 1.000).

Table 7 and Table 8 present the main variables related to MetS, according to gender and ethnicity respectively. Characteristics for most variables across the treatment groups showed no significant differences. However, mean waist size was smaller for females in the intervention (99 cm, SD ±11) versus the control (106 cm, SD ±12), borderline statistical significance (p = 0.055). According to ethnicity, HDL levels were significantly higher for WEs in the intervention versus the control (1.3 mmol/l, IQR 1.1 - 1.8, v.s 1.2 mmol/l, IQR 1.1 - 1.3, p = 0.015) and triglycerides levels were lower (1.4 mmol/l, IQR 0.8 - 1.9, v.s 1.7 mmol/l, IQR 1.4 - 2.2) although statistical significance was borderline (p = 0.053).

**Table 7:** Measures related to metabolic syndrome by gender

Metabolic syndrome components	All		Intervention		Control		p value (I vs. C)	
	Male, (n = 36)	**Female**, (n = 46)	Male, (n = 21)	Female, (n = 21)	Male, (n = 15)	Female, (n = 25)	**Male**,	Female
**Waist circumference(cm)**	109.0 ± 8.7	102.8 ± 11.5	108.5 ± 7.6	99.2 ± 10.5	109.6 ± 10.3	105.8 ± 11.7	p = 0.669	p = 0.055

**Systolic BP (mmHg)**	134.9 ± 13.7	130.5 ± 16.6	135.3 ± 11.7	134.4 ± 14.8	134.3 ± 16.6	127.2 ± 17.5	p = 0.822	p = 0.140

**Diastolic BP (mmHg)**	86.3 ± 9.6	85.5 ± 9.6	86.1 ± 10.6	85.1 ± 9.0	86.6 ± 8.4	85.8 ± 10.2	p = 0.890	p = 0.783

**HDL cholesterol (mmol/l)**	1.15 [1.00 - 1.40]	1.20 [1.10 - 1.50]	1.20 [1.00 - 1.40]	1.30 [1.10 - 1.80]	1.10 [0.90 - 1.50]	1.20 [1.10 - 1.45]	p = 0.528	p = 0.238

**Triglycerides (mmol/l)**	1.50 [1.05 - 2.10]	1.60 [1.20 - 2.00]	1.40 [0.90 - 2.10]	1.60 [1.00 - 1.80]	1.60 [1.20 - 2.20]	1.70 [1.40 - 2.10]	p = 0.541	p = 0.116

**FPG (mmol/l)**	5.29 ± 0.53	5.15 ± 0.57	5.36 ± 0.47	5.11 ± 0.38	5.19 ± 0.61	5.19 ± 0.70	p = 0.339	p = 0.611

**Proportion meeting IDF criteria for:**								
**BP**	31 (86.1)	42 (91.3)	17 (81.0)	20 (95.2)	14 (93.3)	22 (88.0)	p = 0.376	p = 0.614
**FPG**	11 (30.6)	11 (23.9)	8 (38.1)	4 (19.0)	3 (20.0)	7 (28.0)	p = 0.295	p = 0.717
**HDL**	29 (80.6)	43 (93.5)	16 (76.2)	19 (90.5)	13 (86.7)	24 (96.0)	p = 0.674	p = 0.585
**Triglycerides**	32 (88.9)	35 (76.1)	18 (85.7)	17 (81.0)	14 (93.3)	18 (72.0)	p = 0.626	p = 0.717

**Total no of IDF criteria met:**								
**3**	12 (33.3)	13 (28.3)	8 (38.1)	5 (23.8)	4 (26.7)	8 (32.0)	p = 0.903	p = 0.636
**4**	17 (47.2)	27 (58.7)	9 (42.9)	14 (66.7)	8 (53.3)	13 (52.0)		
**5**	7 (19.4)	6 (13.0)	4 (19.0)	2 (9.5)	3 (20.0)	4 (16.0)		

Parametric continuous data are expressed as mean ± S.D; nonparametric continuous data as median [IQR]; categorical data as number (%).

BP (blood pressure); **HDL **(high density lipoprotein); **FPG **(fasting plasma glucose); **IDF **(International Diabetes Federation).

**Table 8:** Measures related to metabolic syndrome by ethnicity (South Asian and white European only*)

Metabolic syndrome components	All		Intervention		Control		p value (I vs. C)	
	**SA, (n = 18)**	**WE, (n = 62)**	**SA, (n = 11)**	**WE, (n = 31)**	**SA, (n = 7)**	**WE, (n = 31)**	**SA**	**WE**

**Waist circumference(cm)**	101.6 ± 10.6	106.8 ± 10.8	101.6 ± 8.4	104.7 ± 10.8	101.6 ± 14.1	109.0 ± 10.5	p = 0.989	p = 0.118

**Systolic BP (mmHg)**	135.2 ± 16.8	132.0 ± 15.1	137.6 ± 15.4	133.9 ± 12.4	131.3 ± 19.4	130.1 ± 17.5	p = 0.452	p = 0.326

**Diastolic BP (mmHg)**	90.2 ± 9.7	84.8 ± 9.3	92.6 ± 8.1	83.1 ± 9.1	86.6 ± 11.5	86.4 ± 9.3	p = 0.213	p = 0.163

**HDL cholesterol (mmol/l)**	1.20 [0.98 - 1.43]	1.20 [1.08 - 1.43]	1.10 [0.90 - 1.20]	1.30 [1.10 - 1.80]	1.30 [1.20 - 1.60]	1.20 [1.10 - 1.30]	p = 0.082	p = 0.015

**Triglycerides (mmol/l)**	1.65 [1.20 - 1.95]	1.60 [1.10 - 2.03]	1.70 [1.20 - 2.10]	1.40 [0.80 - 1.90]	1.60 [1.40 - 1.90]	1.70 [1.40 - 2.20]	p = 0.784	p = 0.053

**FPG (mmol/l)**	5.29 ± 0.60	5.20 ± 0.55	5.20 ± 0.49	5.25 ± 0.43	5.43 ± 0.77	5.15 ± 0.65	p = 0.450	p = 0.480

**Proportion meeting IDF criteria for:**								
**BP**	15 (83.3)	56 (90.3)	9 (81.8)	28 (90.3)	6 (85.7)	28 (90.3)	p = 1.000	p = 1.000
**FPG**	5 (27.8)	17 (27.4)	3 (27.3)	9 (29.0)	2 (28.6)	8 (25.8)	p = 1.000	p = 1.000
**HDL**	14 (77.8)	56 (90.3)	8 (72.7)	27 (87.1)	6 (85.7)	29 (93.5)	p = 1.000	p = 0.671
**Triglycerides**	13 (72.2)	52 (83.9)	7 (63.6)	28 (90.3)	6 (85.7)	24 (77.4)	p = 0.596	p = 0.300

**Total no of IDF criteria met:**								
**3**	8 (44.4)	17 (27.4)	6 (54.5)	7 (22.6)	2 (28.6)	10 (32.3)	p = 0.446	p = 0.670
**4**	9 (50.0)	33 (53.2)	5 (45.5)	18 (58.1)	4(57.1)	15 (48.4)		
**5**	1 (5.6)	12 (19.4)	0 (00.0)	6 (19.4)	1 (14.3)	6 (19.4)		

## Discussion

As far as we are aware, the TRIMS study is the first RCT in the UK to investigate the delivery of a lifestyle intervention to people with MetS to improve CVD and diabetes risk factors. More, specifically, we have developed an evidence based structured group education programme and aim to determine the impact of attending the education programme on features of MetS and quality of life after 12 months of follow-up.

Existing evidence from clinical trials is limited regarding the effectiveness of strategies aimed at primary prevention of T2DM and/or CVD in people with MetS. However, an RCT conducted in Italy in people with MetS demonstrated that an intervention promoting a Mediterranean style diet significantly reduced the prevalence of MetS after 2 years of follow-up compared to the control group [53]. Findings from another Italian study with 1 year of follow-up suggest that a lifestyle intervention focusing on nutrition and physical activity is effective in both reversing MetS and reducing the incidence of diabetes [54]. Additionally, a recent sub-group analysis of a larger RCT in a Norwegian population, found that a combined diet and exercise intervention in people with MetS significantly reduced the prevalence of MetS at 1 year follow-up compared to the control group [52]. Further evidence from secondary data analyses of two large diabetes prevention programmes, the Diabetes Prevention Program (DPP) in the US and the Finish Diabetes Prevention Study (DPS), suggests that an intensive lifestyle intervention focused on weight loss and exercise significantly reduces the prevalence of MetS [55,56]. In the DPP, lifestyle was compared to both placebo and metformin [55] and in the DPS lifestyle was compared to usual care [56]. However, the populations studied were highly selected, in that all had impaired glucose tolerance. Research is needed into the effectiveness of lifestyle programmes for primary prevention of T2DM and CVD in mixed-ethnic populations with MetS. The TRIMS programme was developed as a pragmatic early intervention that can be easily implemented to a large number of people in primary care.

Results from the analysis of baseline data indicate that the randomisation procedure led to balanced samples between the study arms, with no statistically significant differences in the characteristics between the overall intervention and control groups. When the main variables related to MetS were compared separately by ethnicity and gender, one variable showed a significant difference between the intervention and control group, and two were of borderline statistical significance. However, these observations should not be over interpreted; as significance was assessed at the 5% level, some statistical differences might have been expected by chance due to the number of variables compared.

We acknowledge that our intervention is less intensive than the diet and exercise intervention in the ODES trial [52], on which our power calculation is based. However, our intervention is pragmatic and designed to be appropriate to be delivered in the "real world setting". Additionally, we are not looking for a difference of 55% as found in the ODES trial but a more modest difference of 30% between our intervention and control groups. We consider that the intensity of our intervention is sufficient to justify using resolution of MetS as the primary outcome. Furthermore, evidence from our random effects meta-analysis we conducted suggests that lifestyle advice (diet and/or exercise) is effective for reversing MetS (OR 4.15, 95% CI 3.92-4.39) [31].

Additionally, if the prevalence of MetS is in the region of 30% [13], one-to-one counselling is unlikely to be feasible in a primary care setting. A previous study, conducted by our research group in people with impaired glucose tolerance (PREPARE) [27] was found to be very effective at improving glycaemic control and increasing physical activity (in particular walking) alongside only 3 hours of contact time. We have taken a pragmatic approach and developed a group based education programme, with a written evidence based curriculum, underpinned with appropriate learning and health-behaviour theories, and providing 6 hours of contact time.

Around half of all participants were prescribed a statin and/or antihypertensive at baseline. However, this was similar for both intervention and control group participants. We acknowledge that compliance could change during the study period but this could be a possible benefit of the intervention. In addition, it is recognised that prescribing may change, including new prescriptions for lipid or blood pressure medication to those who were medication naïve at baseline. This would then result in no change in the appropriate MetS criterion. However, prescribing changes will occur in a "real world setting" and are likely to occur in both groups. We are recording prescription medications at baseline, 6 months and 12 months and we will also consider looking at prescription records at the end of the study. Furthermore, our planned analysis using multi-variable regression methods will allow us to consider adjustment for prescribed medication.

We recognise that the majority of previous epidemiological studies and clinical trials have used the NCEP definition [5,37] to identify MetS. However, bearing in mind the ethnic diversity in our study population, provision of ethnic-specific cut-off points for waist circumference and central obesity within the IDF definition [6] led to preferential selection of this definition for our trial. Additionally, secondary outcome measures for the study include the prevalence of MetS according to NCEP criteria at 12 months follow-up compared to baseline.

We acknowledge that our study excluded people who were unable to understand, speak and read English. However, our study population includes a subset of the SA population in the UK (English speaking people mainly of Indian origin), and overall 22% of participants were of SA ethnicity. Additionally, we took steps to address cultural needs, for example by including foods commonly eaten in the SA community. If the education programme is found to be successful we will consider adapting it for non-English speakers and ethnically diverse populations. However, we acknowledge that additional work would need to be conducted to test for transferability of the intervention to other settings and populations.

## Conclusions

As far as we are aware, the TRIMS study is the first RCT in the UK to investigate the delivery of a structured group education programme to individuals with MetS to improve management of cardiovascular and diabetes risk factors. Follow-up data will be collected at 6 and 12 months. Results will provide important evidence to help inform primary prevention strategies for diabetes and CVD in high risk individuals, in a multi-ethnic population.

## Ethical approval

The study is being conducted in accordance with the approvals granted by the Leicestershire, Northamptonshire and Rutland Research Ethics Committee, University Hospitals of Leicester NHS Trust, NHS Leicester City, and NHS Leicestershire County and Rutland.

## Abbreviations

BMI: Body Mass Index; BP: Blood Pressure; C: Control group; CVD: Cardiovascular disease; DINE: Dietary Instrument for Nutrition Education; DPP: Diabetes Prevention Programme; DPS: Finish Diabetes Prevention Study; EQ-5D: EuroQol EQ-5D questionnaire; FPG: Fasting Plasma Glucose; GP: General Practitioner; GSE: General Self Efficacy scale; HADS: Hospital Anxiety and Depression Score; HbA1c: Glycated haemoglobin; HDL: High Density Lipoprotein cholesterol; hs:-CRP High-sensitivity-C-reactive protein; I: Intervention group; IDF: International Diabetes Federation; IMD: Indices of Multiple Deprivation; IPAQ: International Physical Activity Questionnaire; IQR: Interquartile range; LDL: Low Density Lipoprotein cholesterol; MetS: Metabolic Syndrome; MRC: Medical Research Council; NS-SEC-5-class: National Statistics Socio-economic Classification 5-class version; ODES: Oslo Diet and Exercise Study; OGTT: Oral Glucose Tolerance Test; RCT: Randomised Controlled Trial; SA South Asian; T2DM: Type-2 diabetes; TRIMS: The Reversal Intervention for Metabolic Syndrome; VAS: Visual Analogue Scale; WE: White European.

## Conflict of interests

KK and MJD have received sponsorship for attending conferences and honorariums from pharmaceutical companies that manufacture drugs for hyperglycaemia and anti-obesity drugs. AJD has received sponsorship for attending conferences.

## Authors' contributions

KK is the principal investigator of the study, AJD, MJD, MAS, NT and TY are co-investigators, and JT is an advisor. This work forms part of a PhD that AJD is currently undertaking. AJD and JT were involved with curriculum development and delivery of the education programme. All authors contributed to conception and design of the study. AJD, KK, MJD, MAS and NT contributed to the drafting and critical revision of the manuscript. All authors read and approved the final manuscript.

## Authors' information

AJD: MSc, RGN, Research Associate in Nursing. MJD: MD, FRCP, Professor of Diabetes Medicine. MAS: PhD, Senior Research Fellow. NT: PhD, Research Fellow in Medical Statistics. JT: MSc, SRD, Advanced Diabetes Practitioner. TY: PhD, Research Associate. KK: PhD, MD, FRCGP, FRCP, Professor of Primary Care Diabetes & Vascular Medicine.
